# The Human Amniotic Mesenchymal Stem Cells (hAMSCs) Improve the Implant Osseointegration and Bone Regeneration in Maxillary Sinus Floor Elevation in Rabbits

**DOI:** 10.1155/2019/9845497

**Published:** 2019-12-11

**Authors:** Lu Yin, Zhi-xuan Zhou, Ming Shen, Ning Chen, Fei Jiang, Shou-Lin Wang

**Affiliations:** ^1^Jiangsu Key Laboratory of Oral Diseases, Nanjing Medical University, No. 140, Han Zhong Road, Nanjing 210029, China; ^2^School of Public Health, Center for Global Health, Nanjing Medical University, No. 101 Longmian Av., Nanjing 211166, China; ^3^Department of Polyclinic, Affiliated Hospital of Stomatology, Nanjing Medical University, No. 136, Han Zhong Road, Nanjing 210029, China; ^4^Department of Dental Implant, Affiliated Hospital of Stomatology, Nanjing Medical University, No. 136, Han Zhong Road, Nanjing 210029, China

## Abstract

Insufficient bone height in the posterior maxilla is a challenging problem in dental implantation. Bio-Oss, though routinely used in maxillary sinus floor elevation (MSFE), is not osteoinductive. Human amniotic mesenchymal cells (hAMSCs) isolated from placental tissues have potential for multidifferentiation and immunomodulatory properties and can be easily obtained without the need for invasive procedures and without ethical concerns. This is the first study to use hAMSCs to improve implant osseointegration and bone regeneration after MSFE. Human AMSCs were loaded into a fibrin gel and injected into rabbit MSFE models. The rabbits were assigned to four groups (*n* = 3 per group), i.e., the control group, the hAMSC group, the Bio-Oss group, and the hAMSC/Bio-Oss group. The animals were sacrificed at postsurgery for four and twelve weeks and evaluated by histology and immunohistochemistry. Bone volume, bone volume/tissue volume, bone-to-implant contact ratio, and vessel-like structures in the hAMSC/Bio-Oss group were significantly better than those in other groups in the peri-implant and augmented areas. Immunofluorescence staining showed that alkaline phosphatase (ALP) activities of two hAMSC groups were higher than those of the other two groups. Sequential fluorescent labeling was performed in all of the 12-week groups. Observations showed that hAMSCs accelerated mineralized deposition rates on implant surfaces and in bone-augmented areas. These data demonstrated that hAMSCs could enhance implant osseointegration and bone regeneration after MSFE and might be used to optimize dental implantation in the future.

## 1. Introduction

Severely insufficient bone volume in the posterior maxilla (bone height < 3 mm) is a commonly encountered clinical problem after patients' tooth loss, which seriously affects the quality of patients' life. Maxillary sinus floor elevation (MSFE) is a routine surgical procedure to increase bone height in the posterior maxilla [1]. Typically, in this procedure, bone grafting substitutes and/or biomaterials are filled on the sinus floor for the purpose of improving the initial stability of the implant and bone augmentation in MSFE [2]. Bio-Oss (Bio-Oss®, Geistlich Biomaterials, Wolhusen, Switzerland), which has similar properties to human bone, is a most commonly used bone grafting substitute in periodontal surgery, alveolar surgery, and dental implantation [3]. However, Bio-Oss has been reported to lack an intrinsic osteoinductive capacity and works merely as a scaffold in MSFE. Bio-Oss induces neither bone regeneration nor implant osseointegration and might even delay early bone formation after MSFE [4–6]. Thus, a search for new strategies to improve and optimize clinical outcomes and implant osseointegration in MSFE is urgently needed.

The osteogenic effect of Bio-Oss is enhanced by mixing autologous bone during the MSFE; however, the acquisition of additional autogenous bone is both invasive and risky. Many researchers have tried to apply tissue engineering strategies to promote bone regeneration in MSFE. It was reported that calcium phosphate scaffolds loaded with mesenchymal stem cells (MSCs) could be used in MSFE to reach the desired osteogenic effect [7–9]. MSCs that are derived from the bone marrow or adipose tissue are obtained by invasive procedures, and their stem cell characteristics are impacted by the disease stage and age of the donors [10]. Thus, the search for other suitable stem cells that can be isolated noninvasively and which display superior proliferative and differentiation capacities is also urgently needed.

Human amniotic mesenchymal stem cells (hAMSCs) possess a greater potential for proliferation and differentiation and can be obtained from the discarded placenta and then be easily isolated without any invasive procedures or ethical controversies [11, 12]. Furthermore, hAMSCs show decreased immunogenicity and thus hold great promise for clinical applications [13–16]. hAMSCs have been successfully applied to repair rabbit cartilage defects, rat spinal cord injury, and even mouse lung tissue and liver tissue fibrosis [15, 17–20]. Thus, the potential of applying hAMSCs in MSFE has not been verified. We hypothesized that Bio-Oss in combination with hAMSCs could be applied in the setting of MSFE and could therefore optimize implant osseointegration and regeneration in bone augmentation areas and reduce the period of post-operative healing after MSFE.

## 2. Materials and Methods

### 2.1. Isolation, Cultures, and Identification of hAMSC

Human term placentas of normal pregnancies (ranging from 38 to 41 weeks) were obtained after cesarean section. The study protocol was approved by the Ethics Committee of Nanjing Medical University (No. PJ2014-079-001), and all women had signed written informed consent. We followed a previously established protocol to isolate hAMSCs [21]. Briefly, the fresh amnions were cut into small pieces and then separated and washed in phosphate-buffered saline (PBS) containing 100 U/ml penicillin and 100 *μ*g/ml streptomycin (Beyotime, China). Amnion fragments were digested for 10 minutes at 37°C in PBS containing 2.4 U/ml dispase (Roche, Germany). The incubated fragments were transferred to complete medium that contained *α*-MEM (Gibco, USA) supplemented with 10 percent heat-inactivated fetal bovine serum (FBS; Gibco, USA) to rest the tissue and cell suspension for 5 minutes at room temperature. After the resting period, the fragments were immersed in complete medium with 0.75 mg/ml collagenase (Roche, Germany) for approximately 3 hours at 37°C. Amnion fragments were then removed. Mobilized cells were passed through a 100 *μ*m cell strainer (BD Falcon, USA), and the cells were collected by centrifugation at 300 × *g* for 10 minutes. Cells were cultured in *α*-MEM and used before personal passage three. Cell morphology was constantly observed, and photographs were taken for record.

### 2.2. Detection of hAMSC Surface Markers by Flow Cytometry Analysis

Human AMSCs were trypsinized and resuspended in PBS with fluorescein-conjugated primary antibodies. The samples were incubated for 30 minutes at 1 : 200 dilution and incubated in a dark place. After centrifuging and washing with PBS, the samples were analyzed on a FACSCalibur flow cytometer (BD Biosciences, USA). APC-conjugated CD105 and CD29 primary antibodies, Cy3-conjugated CD90 primary antibody, PE-conjugated CD34 primary antibody, and FITC-conjugated human leukocyte antigen-DR (HLA-DR) primary antibody were purchased from Miltenyi Biotec (Germany).

### 2.3. Identification of Multidifferentiation Potential of hAMSCs

Human AMSCs were induced to differentiate into adipocytes, osteoblasts, and chondroblasts in vitro following the protocols previously described [21].

To induce osteogenic differentiation, hAMSCs were cultured in DMEM (Gibco, USA) that was supplemented with 10% FBS, 10 mM *β*-glycerophosphate, 0.2 mM ascorbic acid, and 10^−8^ M dexamethasone (Sigma, USA). Cells were cultured for 21 days, and the medium was replaced every three days. To demonstrate osteogenic differentiation, the cultures were fixed in 4% PFA and stained by Alizarin Red S solution.

To induce adipocyte differentiation, hAMSCs were cultured in DMEM that was supplemented with 10% FBS, 0.5 mM IBMX (Sigma, USA), 200 *μ*M indomethacin (Sigma, USA), 10^−6^ M dexamethasone, and 10 *μ*g/ml insulin (Sigma, USA) in chamber slides (NUNC, USA). The cells were cultured, and the medium was replaced every 2-3 days. After 21 days of culture, the cells contained neutral lipids in fat vacuoles and were then fixed in 4% PFA and stained by fresh Oil Red O solution (Sigma, USA).

To induce chondrogenic differentiation, aliquots of hAMSCs were pelleted in polypropylene conical tubes in 0.5 ml of DMEM containing 6.25 *μ*g/ml insulin, 6.25 *μ*g/ml transferrin, 6.25 *μ*g/ml selenous acid, 5.33 *μ*g/ml linolenic acid, 1.25 mg/ml BSA, 0.35 mM proline, 1 mM sodium pyruvate, 10^−7^ M dexamethasone, and 0.1 mM L-ascorbic acid-2-phosphate (all were obtained from Sigma, USA) and then supplemented with 10 ng/ml TGF-*β* (R&D Systems, USA). This medium was replaced every three days for 28 days. Pellets were fixed in 4% PFA and stained by Toluidine Blue.

### 2.4. Animal Experiments and Experimental Groups

**Figure 1:**
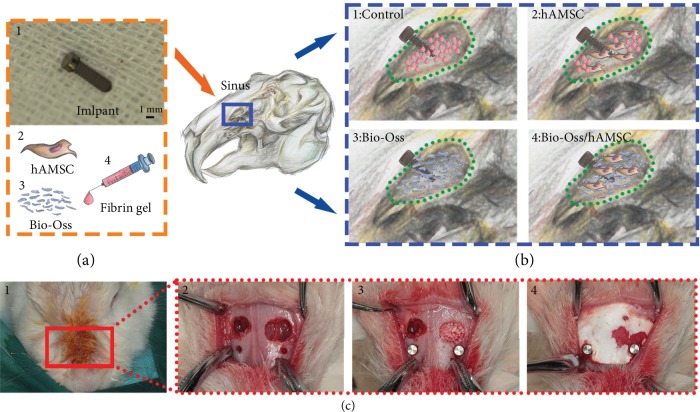
Schematic diagram of rabbit maxillary sinus floor elevation. (a) Implant and filling materials into the sinus: fibrin gel, human amniotic mesenchymal stem cells (hAMSCs), and Bio-Oss (Bio-Oss®, Geistlich Biomaterials, Wolhusen, Switzerland). (b) Four experimental groups. (c) Surgical procedure.

The study was approved by the Institutional Animal Care and Use Committee of Nanjing Medical University (No. PJ2014-079-001). Twelve New Zealand white rabbits were used for the experiments. According to the sinus filling materials, the animals were divided into four groups (*n* = 3 per group): (1) control group as the negative control; (2) hAMSC group; (3) Bio-Oss group as the positive control; and (4) hAMSC/Bio-Oss group (Figure 1(b)).

The animals were anaesthetized by intravenous injection of pentobarbital sodium (1.5 mg/kg). The surgical site was injected subcutaneously with 0.5 ml of 1% lidocaine with epinephrine (1 : 100,000). The nasal bone and nasoincisal suture lines were exposed. The 5 mm diameter circular wall in the nasal bone was removed by a round bur. The sinus membrane was then elevated with a surgical curette. Plasminogen-depleted human fibrinogen solution was prepared by serum-free media at a concentration of 5 mg/ml and filtered for sterility. Human AMSCs were trypsinized and then resuspended in fibrinogen solution at a cell density of 0.5 × 10^6^ hAMSCs per 100 *μ*l. The number of the cells was counted by an automatic cell counter called Countess II (Invitrogen, USA). The fibrinogen-hAMSC solution was catalyzed by thrombin (50 U/ml; Sigma, USA) as 20 : 1 to form the hAMSC-gel. The hAMSC-gel was injected into the sinus before crosslinking for the hAMSC and hAMSC/Bio-Oss groups. In the control group, only fibrin gel was injected into the maxillary sinus. In the Bio-Oss group, the gel and Bio-Oss are mixed and implanted into the maxillary sinus (Figures 1(a) and 1(b)). A 1.5 mm diameter, 5 mm long customized mini-implant (Bioconcept Co., Ltd., China), which was sand-blasted, large grit, and acid etched and has the same surface structure as a conventional implant, was installed below the bone window. The mini-implant has good biocompatibility without systemic toxicity (the test report can be seen in supplementary file). A collagen membrane (Heal-All, China PR) was placed between the maxillary sinus operation area and periosteum. The skin and periosteum were sutured separately (Figure 1(c)). A total of 80,000 U/day of penicillin was injected intramuscularly for 3 days.

### 2.5. Histological and Immunohistochemical Evaluation

The animals were sacrificed by euthanasia after 4 and 12 weeks of the surgical procedure. All specimens were cut parallel to the implant axis in the mesial and distal planes into two parts and the fixed immediately in 10% buffered formalin. Half of the bone blocks were left nondecalcified and embedded in polymethymetacrylate and then subsequently ground and polished to about 40 *μ*m, following which, they were stained with Van Gieson's picro fuchsin (VG) for histology. The other half of the samples were then decalcified, embedded in paraffin, and sliced coronally into 4 *μ*m thick serial sections along the center of the implant position. These decalcified specimens were stained with Masson's trichrome (Beyotime, China) for histology and alpha-smooth muscle actin (*α*-SMA) for immunohistochemistry. Primary antibody targeted to *α*-SMA (ab21027) was purchased from Abcam (UK).

### 2.6. Immunofluorescence Examination

All decalcified specimens in the 4-week and 12-week groups detected the expression of alkaline phosphatase (ALP) by immunofluorescence staining. The primary antibody (anti-ALP 1 : 50, AF2910) was purchased from R&D Systems (USA). The secondary antibody conjugated to Alexa Fluor® 488 (1 : 200, Thermo Fisher, USA) was used. After washing secondary antibody three times by PBS, the cell nuclei were stained by DAPI (1 : 200, Thermo Fisher, USA). When we finished the examination of ALP expression in all specimens, the slices were stained with hematoxylin-eosin (HE) for general histology. The amount of the positive expression was analyzed using a fluorescent microscope (Olympus Corporation, Japan) with higher magnifications (200×) and counted on five random fields for each section by a blinded investigator to experimental design. Data was analyzed by ImageJ software.

### 2.7. Sequential Fluorescent Labeling

A polychrome sequential fluorescent labeling method was carried out to label the mineralized tissue and assess the time course of new bone formation and mineralization. Animals received intraperitoneal injections of the fluorescent dyes in 0.9% saline at four- and eight-weeks postsurgery. Next, 20 mg/kg calcein and 30 mg/kg Alizarin Red (all from Sigma, USA) were used. The sections were examined under a confocal laser scanning microscope (ZEISS, Germany). Excitation/emission wavelengths of the fluorescent dyes were 488/517 nm (calcein) and 543/617 nm (Alizarin Red).

### 2.8. Statistical Analysis

The IBM SPSS version 19.0 statistical software program (SPSS, USA) was used for statistical calculations. Differences between the four groups were analyzed by a one-way analysis of variance (ANOVA) model. A *p* value < 0.05 was considered statistically significant.

## 3. Results and Discussion

### 3.1. Identification of hAMSCs

**Figure 2:**
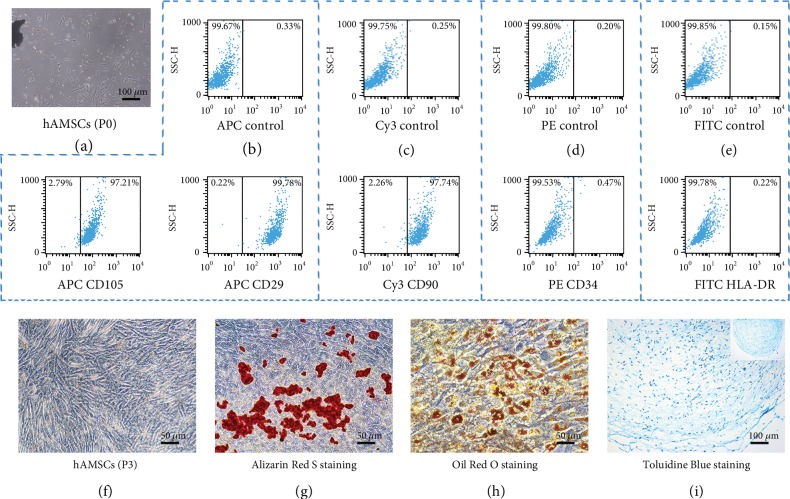
Characterization of human amniotic mesenchymal stem cells (hAMSCs). (a) Primary passage (P0) of hAMSCs. (b–e) Negative controls in eac h flow cytometric histogram. Human AMSCs were positive for CD105 (b), CD29 (b) and CD90 (c), and negative for CD34 (d) and HLA-DR (e). (f) hAMSCs at personal passage three (P3). (G-I) hAMSCs induced by osteogenic (g), adipogenic (h), and chondrogenic (i) media were stained by Alizarin Red S, Oil Red O, and Toluidine Blue solution, respectively.

The hAMSCs obtained by enzyme digestion were found to be adherent within 12 hours. After 24 hours, the majority of the adherent cells stretched and became spindle or short-rod shaped (Figure 2(a)). At personal passage three, hAMSCs showed a fibroblast-like morphology, grew to a confluence of 90 percent, and were arranged in radial or whirlpool patterns (Figure 2(f)).

Flow cytometry showed that hAMSCs expressed representative mesenchymal cell surface markers including CD29 (99.78%), CD105 (97.21%), and CD90 (97.74%) (Figures 2(b) and 2(c)). Human AMSCs were negative for CD34 (0.47%) and HLA-DR (0.22%) expression (Figures 2(d) and 2(e)).

### 3.2. Differentiation Potential of hAMSCs

After 21 days of osteogenic induction, hAMSCs at personal passage three formed many calcium deposits as shown by Alizarin Red S staining (Figure 2(g)). After 21 days of adipogenic induction, cells changed morphological characteristics and we detected accumulation of lipid droplets as shown by Oil Red O staining (Figure 2(h)). To induce chondrogenic differentiation, hAMSCs at personal passage three were digested, centrifuged, and then pelleted and cultured in a chondrogenic induction solution for 28 days to form a cartilage-like sphere. The cartilage-spheres were subjected to cryosectioning and then stained with Toluidine Blue. The blue cartilage matrix was observed in the hAMSC mass (Figure 2(i)).

### 3.3. Rabbit Model Surgical Experiments

In all animals, the surgical procedure was successfully performed, and the wounds healed without any adverse reactions. Animals survived well until every examination time point. Results suggested that hAMSCs were minimally immunogenic in xenogenic transplants.

### 3.4. Histology and Immunohistochemistry

**Figure 3:**
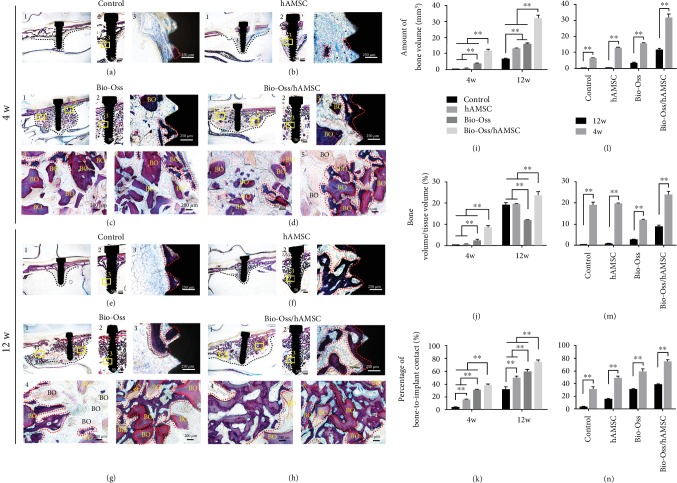
Van Gieson's picro fuchsin (VG) staining. (a–h) VG staining of four groups in the 4- and 12-week groups. All of number 3 were partially enlarged views of number 2. All of numbers 4 and 5 were the partially enlarged views of number 1. (i–n) The amount of bone volume, bone volume/tissue volume, and percent of bone-to-implant contact ratio assessed at the same time points (i–k) and at various time points (l–n). BO: Bio-Oss; red dotted mark range: new bone; ^∗^*p* < 0.05; ^∗∗^*p* < 0.01.

Bone formation was assessed at week 4 and week 12. In the four-week groups, the sinus membrane collapsed into the elevated space and a small new bone filled the threads in the hAMSC group (Figures 3(a) and 3(b)). In the Bio-Oss and hAMSC/Bio-Oss groups, the lifted dome-like space was formed and supported by Bio-Oss and new bone (Figures 3(c) and 3(d)). In all groups, bone formation in the peri-implant area started from the sinus floor and sprouted into the elevated space along the implant surface. In the augmented area, we found new bone formation among Bio-Oss particles that were also derived from the sinus floor in the Bio-Oss and hAMSC/Bio-Oss groups (Figures 3(c) 4, 5 and 3(d) 4, 5). Bone volume (BV), bone volume/tissue volume (BV/TV), and the bone-to-implant contact ratio (BIC) in the hAMSC/Bio-Oss group (11.571 ± 0.871 mm^3^, 8.637 ± 0.650%, and 38.521 ± 0.990%) were significantly higher than those in the control group (0.063 ± 0.005 mm^3^, 0.189 ± 0.015%, and 3.315 ± 0.498%); hAMSC group (0.477 ± 0.067 mm^3^, 0.712 ± 0.100%, and 15.067 ± 1.153%); and Bio-Oss group (3.383 ± 0.573 mm^3^, 2.525 ± 0.428%, and 30.586 ± 1.036%) (*p* < 0.05; Figures 3(i)–3(k)). The results suggested that hAMSC improved bone-to-implant contact and bone regeneration in the early healing period.

Bone formation in all 12-week groups was better than that in the four-week groups, not only in the peri-implant area but also in the augmented area. We showed that the new bone developed into mature parallel-fiber bone and extended towards the apex of the implants and center of the augmented areas in the Bio-Oss/hAMSC group (Figure 3(h) 3–5). Moreover, BV, BV/TV, and BIC (31.779 ± 2.140 mm^3^, 23.720 ± 1.597%, and 74.958 ± 2.173%) were significantly higher than those of the control group (6.391 ± 0.367 mm^3^, 19.083 ± 1.097%, and 31.251 ± 3.770%); the hAMSC group (13.112 ± 0.114 mm^3^, 19.575 ± 0.171%, and 49.382 ± 2.312%); and the Bio-Oss group (15.898 ± 0.370 mm^3^, 11.867 ± 0.276%, and 59.087 ± 4.041%) (*p* < 0.05; Figures 3(i)–3(k)). For each group, we also found that BV, BV/TV, and BIC at 12 weeks were significantly higher than what were found at four weeks (Figures 3(l)–3(n)). These results showed that hAMSCs could play a continuously active role in MSFE.

### 3.5. Blood Vessel Formation at Four and 12 Weeks

**Figure 4:**
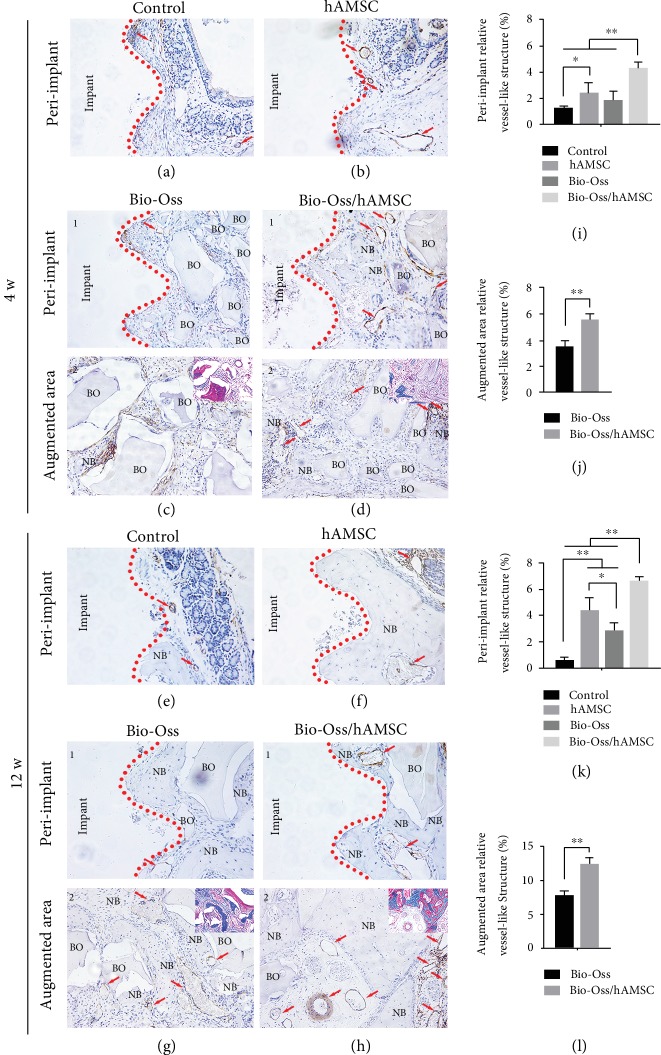
Immunohistochemical staining of *α*-smooth muscle actin (*α*-SMA). (a–h) Four groups in the peri-implant and augmented areas at four and 12 weeks. (i–l) Relative vessel-like structure at four and 12 weeks. BO: Bio-Oss; NB: new bone; red arrow: blood vessel-like structure. Scale bar = 200 *μ*m. ^∗^*p* < 0.05; ^∗∗^*p* < 0.01).

Both at the 4-week and 12-week time points, the vessel-like structures in the Bio-Oss/hAMSC group (4.306 ± 0.419% and 6.675 ± 0.286%) were significantly higher than those in the other three groups in the peri-implant (*p* < 0.05; Figures 4(i) and 4(k)), as well as in the augmented area (5.616 ± 0.454% and 12.367 ± 0.934%) (Figures 4(j) and 4(l)). This result suggested that hAMSC significantly increased capillary tube formation and yielded longer and more mature new blood vessel formation as compared with the other groups.

### 3.6. The Expression of ALP Activity

**Figure 5:**
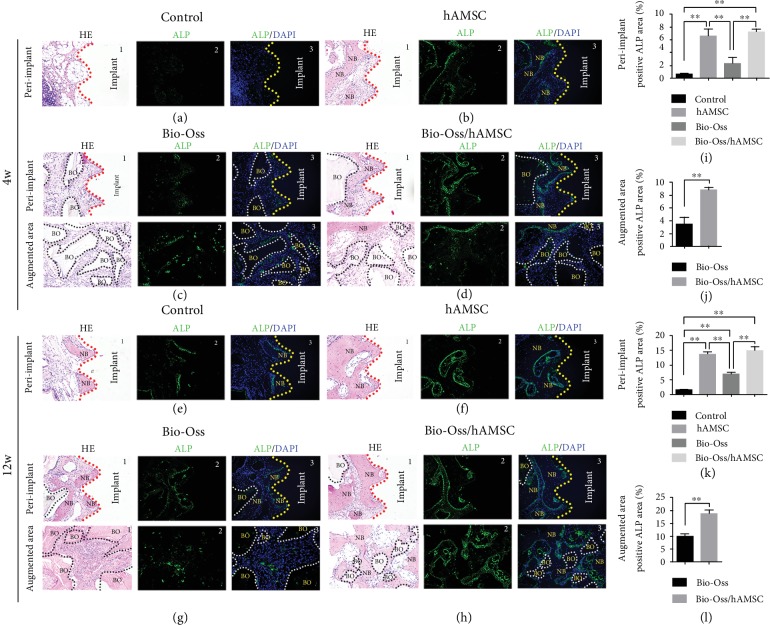
Immunofluorescence of alkaline phosphatase (ALP) and hematoxylin-eosin (HE) staining. (a–h) Four groups in the peri-implant and augmented areas at four and 12 weeks. (i–l) The positive ALP area at four and 12 weeks. DAPI: 4′,6-diamidino-2-phenylindole; BO: Bio-Oss; NB: new bone. Scale bar = 250 *μ*m. ^∗^*p* < 0.05; ^∗∗^*p* < 0.01.

ALP activity usually reflects the degree of osteogenic activity. The immunofluorescence staining showed that the positive ALP areas in the hAMSC group (6.609 ± 1.060%, 13.756 ± 0.532%) and the Bio-Oss/hAMSC group (7.288 ± 0.361%, 14.977 ± 1.223%) were significantly higher than those in the control group (0.669 ± 0.123%, 1.607 ± 0.223%) and Bio-Oss group (2.367 ± 0.851%, 7.026 ± 0.425%) (*p* < 0.05; Figures 5(i) and 5(k)). In the augmented area, the positive ALP areas in the Bio-Oss/hAMSC group (8.799 ± 0.394%, 18.845 ± 1.308%) were also significantly higher than those found in the Bio-Oss group (3.440 ± 1.587%, 9.980 ± 0.770%) (Figures 5(j) and 5(l)). The results revealed that ALP activities in the peri-implant and augmented area in the presence of hAMSCs were higher than the groups without hAMSCs.

### 3.7. Bone Metabolism and Mineralization at Four and Eight Weeks

**Figure 6:**
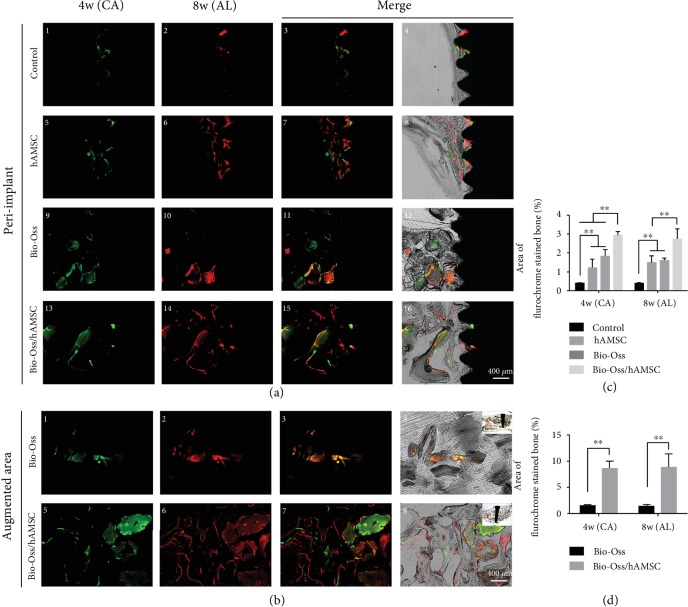
Polychrome sequential fluorescent labeling. (a, b) calcein (CA), Alizarin Red (AL), and merged images in the peri-implant and augmented areas at different time points. Scale bar = 400 *μ*m. (c, d) Relative area of fluorochrome-stained bone in the peri-implant and augmented area. ^∗^*p* < 0.05; ^∗∗^*p* < 0.01.

Dynamic bone histologic analyses were evaluated by fluorescent labeling measurements (Figure 6). The fluorescent labeling showed that the percentages of calcein (CA) and Alizarin Red (AL) positivity in the Bio-Oss/hAMSC group (2.935 ± 0.183%, 2.746 ± 0.534%) were significantly higher than those in the other three groups (*p* < 0.05; Figure 6(c)). In the augmented area, CA and AL in the Bio-Oss/hAMSC group (8.600 ± 1.299%, 8.762 ± 2.523%) were also significantly higher than those found in the Bio-Oss group (1.486 ± 0.195%, 1.444 ± 0.271%) (Figure 6(d)). These results confirmed that hAMSC improved bone formation and accelerated consistent bone mineralization in MSFE and did so especially in the augmented areas.

## 4. Discussion

The implant osseointegration and bone regeneration in the augmentation areas after MSFE directly affected the implant survival rate and long-term outcomes. Bio-Oss is the most commonly used bone grafting material in MSFE and in the severely atrophic maxilla [6]. However, our results and previous reports showed that Bio-Oss has poor osteoinductive properties [6, 9, 22, 23]. In this study, we first applied hAMSCs to improve osteogenesis in MSFE and to remedy poor osteoinductive shortcomings of Bio-Oss. We found that hAMSCs improved implant osseointegration and vascularized bone formation in MSFE.

Some studies showed that several different types of MSC were seeded into the Bio-Oss and then this encouraged favorable osteogenic outcomes in bone augmentation areas [9, 24]. There has not been prior report of hAMSCs promoting osseointegration and bone regeneration in MSFE. This study attempted to apply hAMSCs to improve osteogenesis and implant osseointegration in MSFE. The results of VG staining showed that the application of hAMSCs clearly promoted new bone formation and bone-to-implant contact (Figure 3). Moreover, ALP immunofluorescence staining results also indicated that hAMSC could stimulate the upregulation of ALP expression in the peri-implant and augmented area and display strong osteogenic induction activity (Figure 5). The fluorescent labeling also showed consistent and accelerated bone mineralization, especially in the augmented areas (Figure 6). These results might be attributed to hAMSCs that act as active ingredients for the acceleration of bone metabolism and regeneration in the elevated space after MSFE. Some researchers considered MSCs to participate directly in osteogenesis through differentiation into osteoblasts in the local region [25]. Our results confirmed that hAMSCs could differentiate into osteoblasts (Figure 2); however, we did not find hAMSCs transplanted into the rabbit maxillary sinus following staining with human-specific molecules, such as MHC-II and HLA A+B, in four-week tissue specimens. This seemingly confusing result seemed to be consistent with the reported opinions.

They considered the mechanisms of treating the disease by MSC transplant to be possibly associated with epigenetic modifications that play a crucial role in generating an epigenetic “memory” that maintains physiological and pathological status [26]. In this study, active factors that were secreted by hAMSCs changed the local microenvironment and the status of bone metabolism in the lifted space and not only recruited host stem cells and other precursor cells (e.g., mesenchymal progenitors, osteoblasts, and endothelial cells) but also gave these host cells' epigenetic “memory” to achieve regeneration. This explanation could be supported by *α*-SMA staining (Figures 4(c), 4(d), 4(g), and 4(h)) and fluorescent labeling (Figure 6).

In addition, no obvious inflammatory cell infiltration, adverse effects, or caseous necrosis was observed in the animal specimens from this study. These results suggested that hAMSCs possess both a higher engraftment potential and low immunogenicity in allotransplantation. Many studies have shown that the anti-inflammatory and immunomodulatory capacities of hAMSC are mediated largely through paracrine effects [14, 27–29]. In studies using MSCs to treat lung injury in mice, PCR and immunohistochemical analyses showed that hAMSC intravenous injection could clearly decrease neutrophil infiltration and significantly reduce the expression of IL-1, IL-6, and TNF-*α* in local tissues [18, 19]. Gao et al. transplanted hAMSCs to treat spinal cord injury in rats, and this group reported that hAMSCs reduced myeloperoxidase activity, proinflammatory cytokines (e.g., tumor necrosis factor-alpha, interleukin- (IL-) 1beta, IL-6, IL-17, and interferon-gamma) and cellular apoptosis and increased the levels of anti-inflammatory cytokines (e.g., IL-10 and transforming growth factor-beta1) [20]. In addition, the immunomodulatory characteristics of hAMSC were studied in allogeneic and xenogeneic mixed lymphocyte reactions and their engraftment potential was analyzed by transplantation into neonatal swine and rats [17]. These findings suggest that hAMSCs represent an advantageous type of progenitor cells with potential applications in a variety of cellular therapeutic and transplantation procedures.

## 5. Conclusion

In summary, we applied hAMSCs to remedy the poor osteoinduction experience seen with Bio-Oss and to enhance implant osseointegration and new bone formation after MSFE. Moreover, the immunomodulatory characteristics of hAMSCs make them suitable for allotransplantation. Clearly, clinical trials are required to determine the safety, efficacy, and practical feasibility of the application of hAMSCs. This study demonstrated the potential of hAMSCs in the dental implantation field.

## Data Availability

The data used to support the findings of this study are included within the article.

## Supplementary Materials

Supplementary Materials The implants used in this study were provided by Bioconcept Co., Ltd. (China). The surface treatment methods of the implants were the same as those of current clinical products produced by Bioconcept Co., Ltd. The uploading supplementary PDF file is a certification of the implants without systemic toxicity. This document is an additional answer to reviewers' comments on the relevant tests of the implants used in this study.
