# Supplementary material for: The Human Amniotic Mesenchymal Stem Cells (hAMSCs) Improve the Implant Osseointegration and Bone Regeneration in Maxillary Sinus Floor Elevation in Rabbits
Source: Stem Cells Int. 2019 Dec 11;2019:9845497. doi: 10.1155/2019/9845497 (PMC6927062; doi:10.1155/2019/9845497)
Supplement: Supplementary Materials — The implants used in this study were provided by Bioconcept Co., Ltd. (China). The surface treatment methods of the implants were the same as those of current clinical products produced by Bioconcept Co., Ltd. The uploading supplementary PDF file is a certification of the implants without systemic toxicity. This document is an additional answer to reviewers' comments on the relevant tests of the implants used in this study. [file 9845497.f1.pdf]

TESTING
CNAS L3940

STUDY TITLE

Tests for systemic toxicity (Oral Route)

TEST ARTICLE

Sand-blasted, Large grit, Acid-etched

IDENTIFICATION NO.:

2009-10076

TEST FACILITY:
Sichuan Testing Center for Biomaterials and Medical devices
Biomaterials Building in Sichuan University
29 Wangjiang Road, Chengdu, P.R.China
Zip code: 610064
Tel : +86-28-85412428
Fax: +86-28-85412428
Website: www.safetest.com.cn
E-mail: Standard42@163.com

SPONSOR:
BIO CONCEPT CO., LTD

6th Floor, Unit B, Tianan Industry Park, Xinbei Zone,ChangZhou, Jiangsu, China
Zip code: 213022
Tel : +86-0519-82172266
Fax: +86-0519-85172299
Website:
E-mail:

TABLE OF CONTENTS

Page Number

CERTFICATE OF QUALITY ASSURANCE INSPECTIONS 2
SUMMARY 3
INTRODUCTION 4
MATERIALS 4
METHODS 4
RESULTS 5
CONCLUSION 5
RECORD STORAGE 6

CERTFICATE OF QUALITY ASSURANCE INSPECTIONS

This study was conducted according to our Quality Handbook (the fourth edition, in compliance with ISO/IEC 17025 standard). The study has been performed according to standard operating procedures and standard protocols.

The Quality Assurance Unit conducted inspections on the following data. The findings were reported to the Technical Specialist and Management.

Phase Inspected	Date	Auditor	To Technical Specialist and Management
Test	November 12, 2009	Wen Zou	November 15, 2009
Final Report	November 12, 2009	Tun Yuan	November 20, 2009

Wen Zou
QA Representative: Wen Zou

July 19, 2010
Date:

SUMMARY

The acute systemic toxicity test, based on the standard of ISO 10993-11:2006:Biological evaluation of medical devices-Tests for systemic toxicity, was conducted on the test articles, Sand-blasted, Large grit, Acid-etched, to determine the potential for systemic toxicity. The acute systemic toxicity of the test article was evaluated by the oral route extract liquid at a dose of 50mL/kg. The clinical symptom of toxicity of the test mice was observed and the weight of the each mouse was weighed and recorded daily.

Under the conditions of the study, the toxic symptom was not observed in the test sample group and negative control group. The body weight of the sample and negative control mice were increasing daily.

The result proved that there were not acute toxicity for the article, and met the requirement of the ISO 10993-11:2006 :tests for systemic toxicity.

Study and Supervisory

Personnel: **Heng Du**
Wen Zou

Study Supervisor: Weijing Zhu
Weijing Zhu

Study Director: Jie Liang July 20, 2010
Jie Liang **Date Completed**

This report is authorized by signatures above.

INTRODUCTION

The test article was subjected to acute systemic toxicity study for biocompatibility of materials that based on the standard ISO 10993-11:2006: The test was performed in order to determine the potential for acute systemic toxicity of the test article. The test was begun on November 12, 2009 and observations were concluded on November 15, 2009.

MATERIALS

The following test article characterization data was supplied by the sponsor wherever applicable; it did not apply to confidential conformation. The sponsor was responsible for all test article characterization data.

The sample provided by the sponsor was identified and handled as follows:

Test Article:	Sand-blasted, Large grit, Acid-etched
Identification No.:	2009-10076
State:	Taupe metal, Aseptic Packaged (Fig. 2)
Storage Conditions:	Room temperature, sealed
Preparation:	The whole parts of the sample were used for the extract liquid. The test article extract liquid was prepared under the conditions with 0.2g/mL, 121℃, 1h. The extract medium was normal saline. The extract liquid is colorless and transparent. There was no granule in extracted liquid and the extracted liquid was used immediately after extracted

METHODS

Test system:

Species:	mice (Kunming species)
Source:	Chengdu Dashuo Biotechnology Ltd.
Animals:	Sixteen, 8 per sex
Body weight range:	19.84g-23.93g
Acclimation Period:	3 days
Identification method:	label

Justification of test system:

The mouse is specified as an appropriate animal for evaluating acute systemic toxicity by the current ISO standards. The body weight, the toxicity symptom and the death of the mice were easily observed. The mouse is widely used for this purpose and relative index of the toxicity can be determind.

Animal Management:

Husbandry: Conditions conformed to standard ISO 10993-2:2006: biological evaluation of medical devices Part 2: animal welfare requirements.

Food: Mice diet was provided daily.

Water: Freely available water was delivered through an automatic water system.

Contamination: Reasonably expected contaminations in feed or water supply did not have the potential to influence the outcome of this test.

Housing: Animals were housed in groups in plastic cages suspended identified by a card indicating the lab number, animal numbers, test code, sex, animal code and experimental date.

Environmental: The room temperature was monitored daily. The temperature range for the mice was 20~25℃ in a air-conditioned room.

The room humidity was monitored daily. The humidity range for the mice was 40%-70%.

The light cycle was controlled using an automatic timer (12 hours light, 12 hours dark).

Personnel: associates involved were appropriately qualified and trained.

Facility: our facility is an accredited facility.

Selection: only healthy, previously unused animals were selected.

Oral administered：

Sixteen mice (8 male and 8 female) were randomly divided into two groups: sample group (4 per sex) and control group(4 per sex). The experimented mice were weighted and recorded before the administered. Sample extract liquid was administered by oral route to the mice of sample group with doses of 50mL/kg. Control mice were injected normal saline alone. Each animal was weighted and recorded daily for three days and was observed for toxicity reactions of mice.

RESULTS

Under the conditions of the study, the mice of negative control grew normally and their weight increased along with time and no toxic symptom was observed, the animals of sample group were not observed toxicity symptom and their weight increased daily also (Fig. 1, Table 1)..

CONCLUSION

Under the conditions of this study, the test article: Sand-blasted, Large grit, Acid-etched, showed no acute systemic toxicity and met the requirement of ISO 10993-11:2006 standard.

Results and conclusions just apply to the test articles tested. No further evaluation of these results is made by. Any extrapolation of these data is the responsibility of the sponsor.

RECORD STORAGE

All raw data pertaining to this study and a copy of the final report are to be retained in designated archive Files in our center.

Table 1 The weight change of the test animal.

	sex	Animal number	test day			
			1	2	3	4
negative group	♂	1	23.11	25.38	27.37	29.98
		2	23.36	27.16	29.73	30.93
		3	23.70	26.86	29.29	29.24
		4	22.21	23.43	25.91	26.58
		average	23.10	25.71	28.08	29.18
	♀	1	19.84	22.68	25.37	27.78
		2	20.62	22.85	24.62	29.85
		3	20.52	23.94	25.90	27.71
		4	21.53	23.50	25.64	29.28
		average	20.63	23.24	25.38	28.66
test group 2009-10076	♂	1	22.33	25.36	28.61	28.44
		2	21.92	24.91	27.62	29.37
		3	23.67	25.92	27.87	27.94
		4	23.93	28.12	30.12	30.87
		average	22.96	26.08	28.56	29.16
	♀	1	22.10	23.56	25.83	29.10
		2	20.49	23.21	23.69	27.06
		3	21.36	23.54	25.38	27.48
		4	21.08	22.98	24.30	25.96
		average	21.26	23.32	24.80	27.40

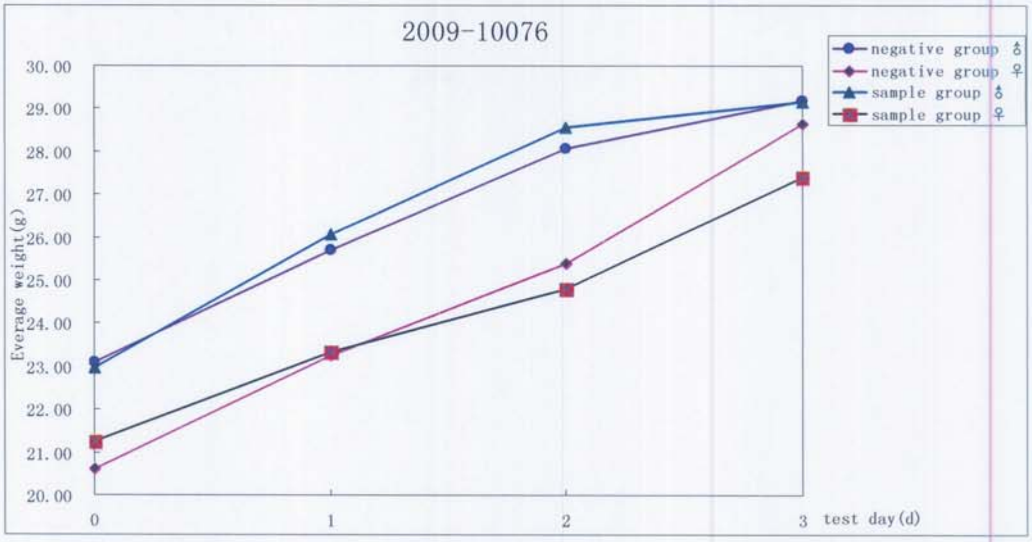

Fig.1 The curve of average weight change of the test animal.

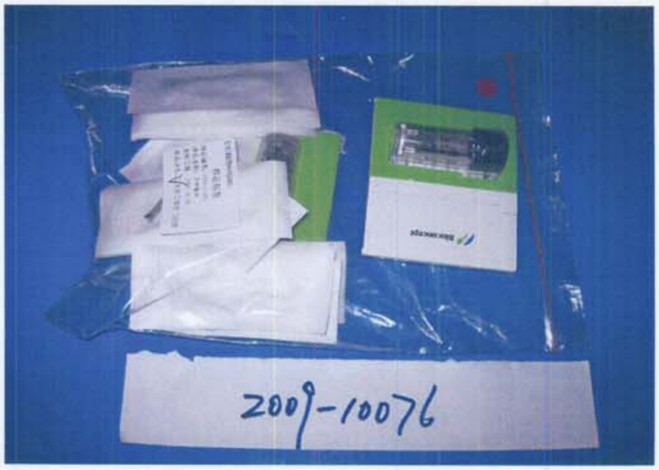

Fig.2 Photograph of the sample
